# Improvement of skin wound healing by giant salamander skin mucus gel wrapped with bone marrow mesenchymal stem cells via affecting integrin family molecules

**DOI:** 10.18632/aging.205792

**Published:** 2024-05-03

**Authors:** Wei Li, Dayong Du, Yan Huang, Cui Xu, Yang Liu, Xiaohong Wu, Jie Yang, Zhipeng Liu, Jianxin Ma, Chaoji Huangfu

**Affiliations:** 1 Department of Cardiology, The 305 Hospital of People’s Liberation Army of China, Beijing 100017, China; 2 Department of Pharmaceutical Sciences, Beijing Institute of Radiation Medicine, Beijing 100850, China; 3 Department of Cadre Ward, The 305 Hospital of People’s Liberation Army of China, Beijing 100017, China; 4 Department of Medical Administration, The 305 Hospital of People's Liberation Army of China, Beijing 100017, China; 5 Department of Neurology, Chengdu Third People’s Hospital, Chengdu 610014, China

**Keywords:** giant salamander skin mucus, bone marrow mesenchymal stem cells, skin, wound healing, integrin

## Abstract

Background: Traditional bandages, gauze, and cotton balls are increasingly insufficient for addressing complex war injuries characterized by severe bleeding and diverse wound conditions. The giant salamander, a species of high medical value, secretes a unique mucus when stimulated, which has potential applications in wound care.

Materials: Giant salamander skin mucus gel dressing wrapped with bone marrow mesenchymal stem cells (BMSCs-GSSM-gel) was prepared and validated. Skin wound injury of rabbit and mouse models were established. Hematoxylin and Eosin, Masson's trichrome, and Sirius red staining were performed. The platelet aggregation rate and coagulation items were measured. Transcriptome sequencing was performed to find potential differential expression genes.

Results: Preparation and characterization of BMSCs-GSSM-gel were performed, and BMSCs-GSSM-gel particles with a diameter of about 200 nm were obtained. BMSCs-GSSM-gel accelerated wound healing in both rabbit and mouse models. BMSCs-GSSM-gel significantly promoted hemostasis via increasing platelet aggregation rate and fibrinogen, but decreasing activated partial thromboplastin time, thrombin time, and prothrombin time. BMSCs-GSSM-gel treatment significantly impacted several genes associated with cell adhesion, inflammatory response, collagen-containing extracellular matrix, and the positive regulation of cell migration based on Gene Ontology (GO) and Kyoto Encyclopedia of Genes and Genomes (KEGG) analysis. Integrin Subunit Beta 4 (ITGB4), Integrin Subunit Alpha 3 (ITGA3), and Laminin Subunit Beta 3 (LAMB3) might be involved in the wound healing process by BMSCs-GSSM-gel.

Conclusions: We proved the BMSCs-GSSM-gel greatly improved the skin wound healing, and it might play a crucial role in the application fields of skin damage repair.

## INTRODUCTION

With the advancement of science and technology and the updating of treatment concepts, traditional bandages, gauze, cotton balls and other general dressings are no longer sufficient to cope with the new situation of war injuries caused by severe bleeding and large and diverse wound conditions [1]. Gel dressings have emerged from the concept of wet wound healing, and are oriented towards multifunctionality, including rapid hemostasis, wound healing support, medicated dressings, and dressings specific to the type of wound [2]. The development of new multifunctional dressings suitable for future wars is necessary to save the lives of the wounded and reduce the incidence of post-traumatic deformities.

The giant salamander is known as a “living fossil” with high medical value. When the salamander encounters external stimuli such as low-voltage electric current or needles, the surface of its body will secrete a kind of mucus [3]. When technicians apply a powder made from the mucus to animal wounds, they find that the wounds heal quickly and automatically, stopping bleeding instantly, and find that there is only a thin line on the surface of the bonded wounds, which are very firmly bonded [3–6]. It was reported that salamander mucus can be used as a surgical adhesive [7]. When the salamander mucus was applied to injured pigskin, a much stronger adhesive, and as flexible as “natural” viscose fibre protein was observed. When the sticky substance was tested on the wounds of live rats, quick skin regeneration ability was achieved [8]. Glu made from salamander mucus is “fully biodegradable and can be easily produced from renewable resources” and, therefore, has the potential for medical applications.

Bone marrow mesenchymal stem cells (BMSCs) are currently the most ideal source of stem cells with multidirectional differentiation and self-renewal capabilities [9]. BMSC can be derived from tissues such as bone marrow and umbilical cord blood, and are capable of differentiating into skin, blood vessels, and adipose tissues. There are no ethical issues associated with their use, and there is minimal immune rejection of allogeneic BMSC transplants [10]. These characteristics have led to widespread interest in the use of BMSC in wound repair, and it has been used in the repair of trauma, burns, and other skin injuries [11, 12]. There are a number of ways to treat skin defects with stem cell transplantation, most of which are based on injections [13]. Several studies applied live cells externally to skin wounds [14]. Clinical trials have also used sprays made from a mixture of BMSCs and fibrin gel, which are applied topically to skin defects [15]. However, these methods are limited by the number of viable stem cells, which leads to less-than-optimal results.

**Figure 1:**
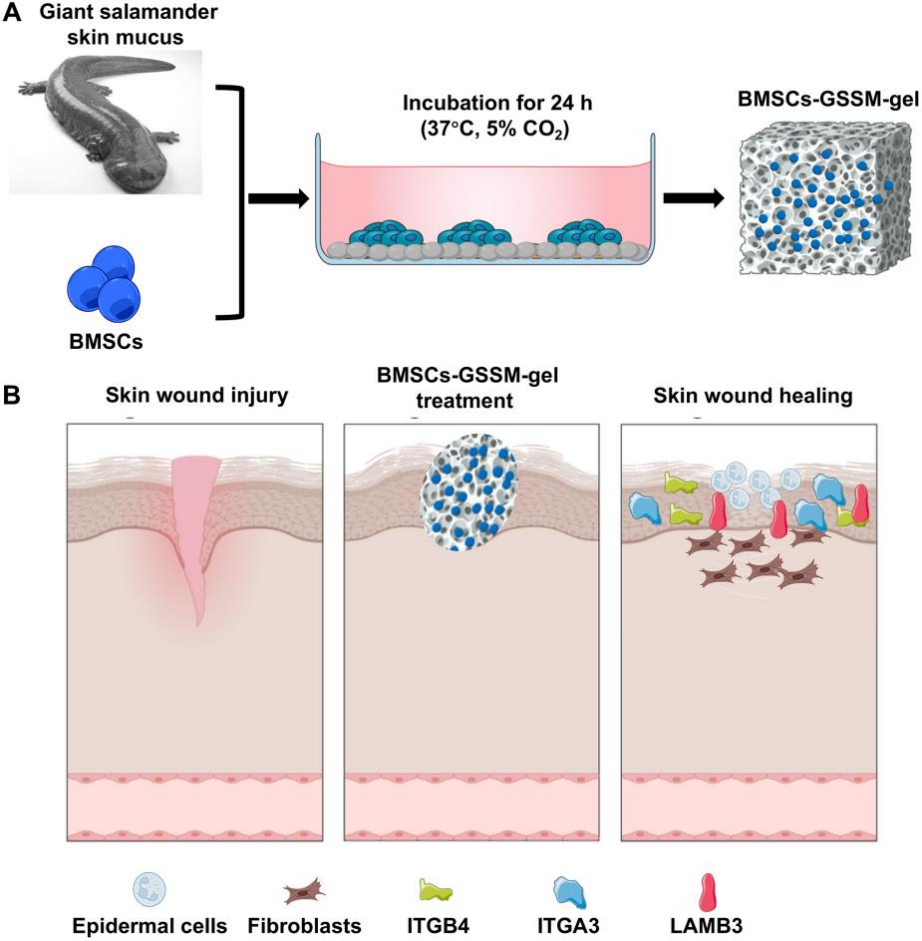
**Schematic diagram of wound healing by BMSCs-GSSM-gel.** (**A**) Preparation process of BMSCs-GSSM-gel. (**B**) Schematic diagram of wound healing.

In order to meet the medical needs of new types of war wounds, the present work is to develop a new type of wound dressing for first aid of war wounds by using salamander skin mucus as the gel matrix, and at the same time loading BMSCs (Figure 1). We demonstrated that BMSCs have good survival and differentiation abilities in the BMSCs-GSSM-gel, and the gel treatment has a significant effect on the improvement of wound healing. Transcriptome sequencing analysis suggested that BMSCs-GSSM-gel might regulate the expression of ITGB4, ITGA3, and LAMB3 to improve skin wound healing. This research provides a detailed theoretical basis for the future preparation and clinical application of BMSCs-GSSM-gel in the field of skin wound healing.

## MATERIALS AND METHODS

### Preparation of GSSM-gel and BMSCs-GSSM-gel

Salamanders were restrained (Shaanxi Hanyuan Biotechnology Co., Ltd, China), and their skin surfaces were cleansed with distilled water before the mucous was gently scraped off using a soft scraper. The mucous was then subjected to vacuum freeze-drying at temperatures ranging from −50°C to −30°C and a vacuum pressure between 5 Pa and 20 Pa for 12 to 48 hours. Subsequent pulverization of the dried secretion was performed using a cryogenic ultramicro crusher to yield fine granules. These granules were dissolved to form a 1 mg/mL solution. For gel preparation, 0.46 g of sodium alginate (2% w/v) was added to 23 mL of deionized water and stirred at 50°C for 30 minutes in a water bath. Additionally, 0.23 g of glycerol (1% w/v) was incorporated and stirred for 8 hours to achieve complete dissolution, resulting in a polymer sol. The polymer sol was then maintained at 37°C, to which 450 μL of the salamander mucous solution was added and stirred for 2 hours to ensure homogeneity. A 0.03 mol/L calcium chloride solution was then introduced to the mucous-containing polymer sol to facilitate cross-linking and form the giant salamander skin mucus gel (GSSM-gel). BMSC cells (1×10^5^/ml, Wuhan Punosai Life Technology Co., Ltd, China) were inoculated on the surface of GSSM-gel onto a 60 mm diameter dish, and cultured in an incubator (37°C, 5% CO_2_) for 24 h. The GSSM-gel wrapped with BMSCs (BMSCs-GSSM-gel) was made and used for the following experiments.

### Hematoxylin and Eosin (H&E) staining

A 1.5 cm × 1.5 cm polydimethylsiloxane scaffold was prepared with GSSM-gel or BMSCs-GSSM-gel. The gels were fixed in 4% paraformaldehyde overnight. The fixed samples were dehydrated with alcohol, embedded in paraffin wax, and allowed to solidify. Sections of 5-10 μm thickness were obtained using a microtome to enhance tissue contrast and subsequently stained with H&E (#C1422, Applygen, China) after dewaxing. The slides were then dehydrated, mounted with a resin medium, and covered with glass coverslips for preservation. The structural and cellular details within the gels were examined using light microscopy.

### Scanning electron microscopy (SEM)

Samples were prepared by fixing the gel in 0.5% glutaraldehyde for 30 minutes, followed by overnight storage in a liquid nitrogen tank. The samples were then freeze-dried, mounted on a clean sample holder, sputter-coated with gold, and examined to discern internal structures.

### Rhodamine staining

BMSCs-GSSM-gels were washed twice with PBS. Rhodamine 123 (#R8030, Solarbio, China) was applied at a working concentration of 50 μL/mL and incubated at 37°C for 5 minutes, with the resultant staining observed under a fluorescence microscope.

### Immunofluorescence staining

For immunofluorescence, cells were fixed with 4% formaldehyde at room temperature for 25 minutes, followed by three 10-minute washes in PBS. Permeabilization was achieved with 0.2% Triton X-100 (#85111, Thermo Fisher Scientific, USA) for 3 minutes, and non-specific binding was blocked using 5% BSA for 20 minutes. The primary antibody was applied overnight at 4°C. Afterward, cells were incubated with the secondary antibody for 2 hours at room temperature, then washed three times with PBS (#10010023, Gibco, USA). The cells were subsequently examined under a fluorescence microscope to visualize staining. The following antibodies were used: Anti-CD31 antibody (#ab182981, 1:800, Abcam, UK), anti-Von Willebrand Factor antibody (#ab6994, 1:800, Abcam, UK), anti-integrin beta 4 antibody (#ab182120, 1:1000, Abcam, UK), anti-Integrin alpha 3 antibody (#ab131055, 1:1000, Abcam, UK), anti-LAMB3 antibody (ab97765, 1:1000, Abcam, UK), anti-Laminin alpha 5 antibody (#ab210957, 1:1000, Abcam, UK). The staining intensity was evaluated with ImageJ Software.

### Hemostatic effect evaluation of BMSCs-GSSM-gel

20 days post-injury, the blood samples were obtained from the artery of the rabbit’s ear and mixed evenly with sodium citrate in a volume ratio of 9:1. The blood samples were centrifuged at 1500 r/min for 15 minutes, and plasma was obtained. The activated partial thromboplastin time, thrombin time, prothrombin time, and fibrinogen were measured with automated coagulation analyzer (Beckman Kurt ACL7000 blood coagulation instrument, USA).

### Animal experiment

Male New Zealand White rabbits (Peking University Experimental Animal Research Center, China) were depilated using a 10% sodium sulfide solution. The skin was disinfected with medical alcohol, and local anesthesia was administered via a 0.5% lidocaine injection. Full-thickness skin incisions approximately 1 cm in diameter were made on either side of the vertebral column to create a dermal wound model. The wounds were dressed with either the BMSCs-GSSM-gel or saline-soaked medical gauze. BMSCs-GSSM-gel or saline-soaked medical gauze was changed once a day. Healing parameters, such as bleeding, redness, swelling, exudate, pruritus, scab formation, and removal, as well as surface flatness and skin elasticity, were assessed on days 4-, 14-, and 20-days post-injury, with digital photography documenting the healing process.

KM mice (Peking University Experimental Animal Research Center, China) were depilated on the dorsal area using a 10% sodium sulfide solution. After disinfection with alcohol, anesthesia was administered via intraperitoneal injection of 1% pentobarbital sodium. Surgical scissors and tweezers were used to make one to two incisions approximately 10 mm in length and 3 mm in depth on the back of the mice. The prepared gel was then applied to the wounds. A physiological saline gauze group served as the control. Wound healing was observed at 2 minutes, 2 hours, and 2 days after the application of the gel.

### Masson’s trichrome staining

For nuclear staining, sections were treated with hematoxylin for 5 minutes, differentiated in 1% hydrochloric acid alcohol for 3 seconds, and rinsed under running water for 3 minutes. Acid fuchsin was applied for 5 minutes, followed by a 5-minute incubation in phosphomolybdic-phosphotungstic acid. The sections were then stained with aniline blue for 5 minutes and differentiated with 1% glacial acetic acid for 1 minute. After dehydration, slides were mounted with neutral gum and observed with an inverted Olympus microscope.

### Sirius red staining

Sections underwent a sequential immersion in xylene (twice, 20 minutes each), anhydrous ethanol (twice, 10 minutes each), graded alcohols (95%, 90%, 80%, and 70%, each for five minutes), and were then rinsed with distilled water. Staining with saturated picric acid containing Sirius Red was conducted for eight minutes. The sections were dehydrated in anhydrous alcohol, dried at 60°C, cleared in xylene for five minutes, and sealed with neutral gum. Microscopic analysis followed, including image capture and assessment.

### Transcriptome sequencing

The transcriptome sequencing was performed by Shenzhen Weikemeng Technology Group Co., Ltd (China). Briefly, the integrity of the RNA samples and the presence of any DNA contamination were analyzed through agarose gel electrophoresis. RNA purity was assessed using a NanoPhotometer spectrophotometer, while RNA integrity was precisely measured using an Agilent 2100 bioanalyzer. The starting RNA for library construction was total RNA, with a total amount of ≥1 ug. The library construction kit used was the NEBNext^®^ Ultra™ RNA Library Prep Kit for Illumina. Following library construction, the Qubit 2.0 Fluorometer was employed for preliminary quantification, diluting the library to 1.5 ng/ul. Subsequently, the Agilent 2100 bioanalyzer was utilized to assess the insert size of the library; once the insert size met expectations, the effective concentration of the library was accurately quantified using qRT-PCR. Different libraries were pooled according to their effective concentration and the desired output data volume for sequencing on an Illumina platform, generating 150 bp paired-end reads. The fundamental principle of sequencing was Sequencing by Synthesis. In the flow cell of the sequencer, four types of fluorescently-labeled dNTPs, DNA polymerase, and adapter primers were added for amplification. During the extension of the complementary chain in each sequencing cluster, the incorporation of a fluorescently-labeled dNTP released the corresponding fluorescence. The sequencer captured this fluorescent signal, which was then converted into sequencing peaks by computer software, thereby obtaining the sequence information of the fragment to be tested. New gene prediction was performed using StringTie (version 1.3.3b). StringTie was utilized to calculate the read count mapped to each gene. The FPKM for each gene was then computed based on the gene's length, along with the read count mapped to that gene. Differential expression analysis between two comparison groups was conducted using DESeq2 software. The *P*-values were adjusted using the Benjamini & Hochberg method. The adjusted *P*-values and the |log2foldchange| were used as the threshold for significant differential expression. GO enrichment analysis of differentially expressed genes was carried out using cluster-Profiler software, which corrected for gene length bias.

### Western blotting

Tissue samples were pulverized in liquid nitrogen and lysed in RIPA buffer (#R0278, Sigma, USA) containing 1% PMSF. Proteins were resolved via SDS-PAGE and transferred onto PVDF membranes (#GVWP02500, Millipore, USA). Membranes were incubated with primary antibodies overnight at 4°C, followed by secondary antibodies. Protein bands were visualized using chemiluminescence with Thermo ECL Substrate (#32132, Thermo Fisher Scientific, USA) and quantified using ImageJ software. The antibodies used in this research were presented in part 2.6.

### CCK8 assay

For the CCK-8 assay, cells were plated at a density of 2 × 10^3^ cells per well in 96-well plates either onto BMSCs-GSSM-gel or not. After incubation for 24 h, cells were washed with PBS and incubated with CCK8 reagent (#E1008-3000, Applygen, China) at 37°C for four hours. Absorbance at 570 nm was measured using a microplate reader.

### Wound healing assay

The wound healing assay was employed to evaluate cellular migration. Cells were cultured at a density of 4 × 10^5^ per well in six-well plates onto BMSCs-GSSM-gel or not, and grown in serum-depleted media to reach 80% confluence. A controlled scratch was then introduced to the confluent monolayer using a sterile 1 mL pipette tip. Photomicrographs of the wound area were taken at the initial time (0 hours) and after 24 hours using an inverted microscope from Olympus Corporation.

### RT-PCR

RNA from skin tissues was extracted using TRIzol (#15596026, Invitrogen, USA). cDNA was synthesized with 1 mg of total RNA via reverse transcription. Then, the synthesized DNA (10 ng) was subjected to CFX96 cycler (Bio-Rad, Hercules, CA, USA) using SYBR Green Realtime PCR Master Mix (#204243, Qiagen, USA). The quantification of gene expression was normalized utilizing the expression of GAPDH as a reference gene. The relative quantification of mRNA expression was assessed using ΔΔCt method.

### Statistical analyses

Statistical analyses and graphing were performed using Prism Graph Pad 6 software, and data were expressed as Mean ± SEM, and statistically analyzed by one-way ANOVA or Student’s *t*-test, with *P* < 0.05 being considered significant.

### Availability of data and materials

All data generated or analyzed during this study are included in this published article.

## RESULTS

### Preparation and characterization of BMSCs-GSSM-gel

**Figure 2:**
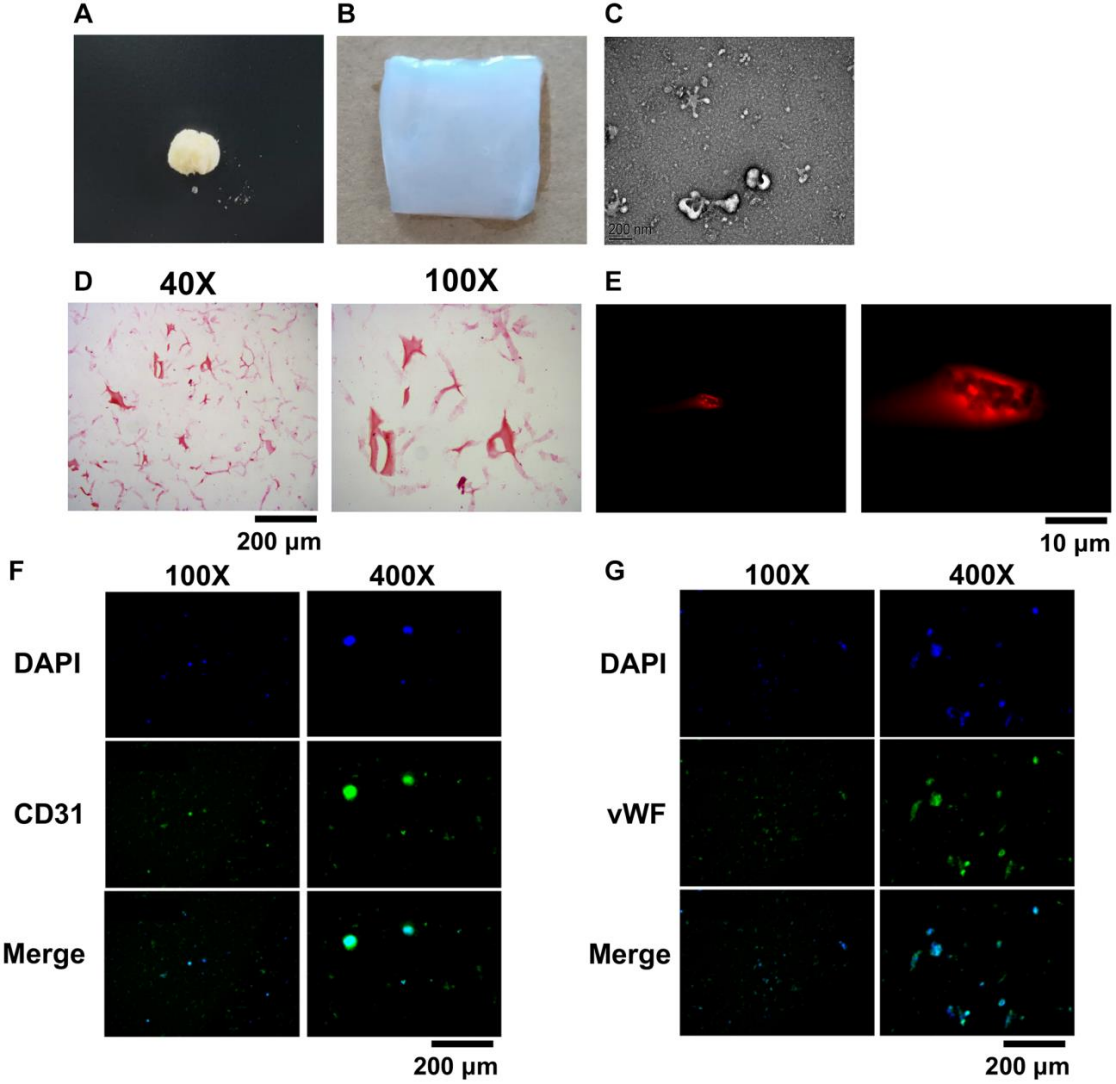
**Preparation and characterization of BMSCs-GSSM-gel.** (**A**) Freeze-dried and crushed skin mucus of salamanders. (**B**) The BMSCs-GSSM-gel was prepared. (**C**) Size observation of BMSCs-GSSM-gel with SEM. (**D**) HE staining was performed to investigate BMSCs-GSSM-gel. (**E**) Rhodamine staining was performed to investigate BMSCs-GSSM-gel. (**F**, **G**) BMSCs differentiation was evaluated by observing CD31 and vWF.

When the skin of giant salamander is stimulated to a certain degree, it will secrete some white mucus, the main components of which are protein, amino acid, mucopolysaccharide and antimicrobial peptide [4]. According to the folk experience, these mucus powders have a remarkable effect on wounds and burns [3]. The group used scratching and stimulation to collect the salamander skin mucus, vacuum freeze-dried it, and crushed the salamander skin secretion with a low-temperature ultra micro crusher to get tiny granular salamander secretion after crushing (Figure 2A). Then, the salamander skin secretion was dissolved, and sodium alginate, glycerol, and calcium chloride solution were added and stirred to cross-link to obtain the hydrogel encapsulating the salamander skin mucus (Figure 2B). Hydrogel particles with a diameter of about 200 nm were obtained (Figure 2C). The heavy red coloring in Figure 2D is the BMSCs in the hydrogel, which shows that the BMSCs can survive well in the BMSCs-GSSM-gel. The results showed that the rhodamine-stained BMSCs showed red fluorescence, suggesting that the cells were more active inside the gel (Figure 2E). CD31 and vWF were used to observe the differentiation ability of BMSCs in the gel. We found that BMSCs in the gel were positive for CD31 and vWF when observed under a laser confocal microscope (Figure 2F, 2G), suggesting that BMSCs had a strong differentiation ability in the gel.

### BMSCs-GSSM-gel accelerated wound healing

**Figure 3:**
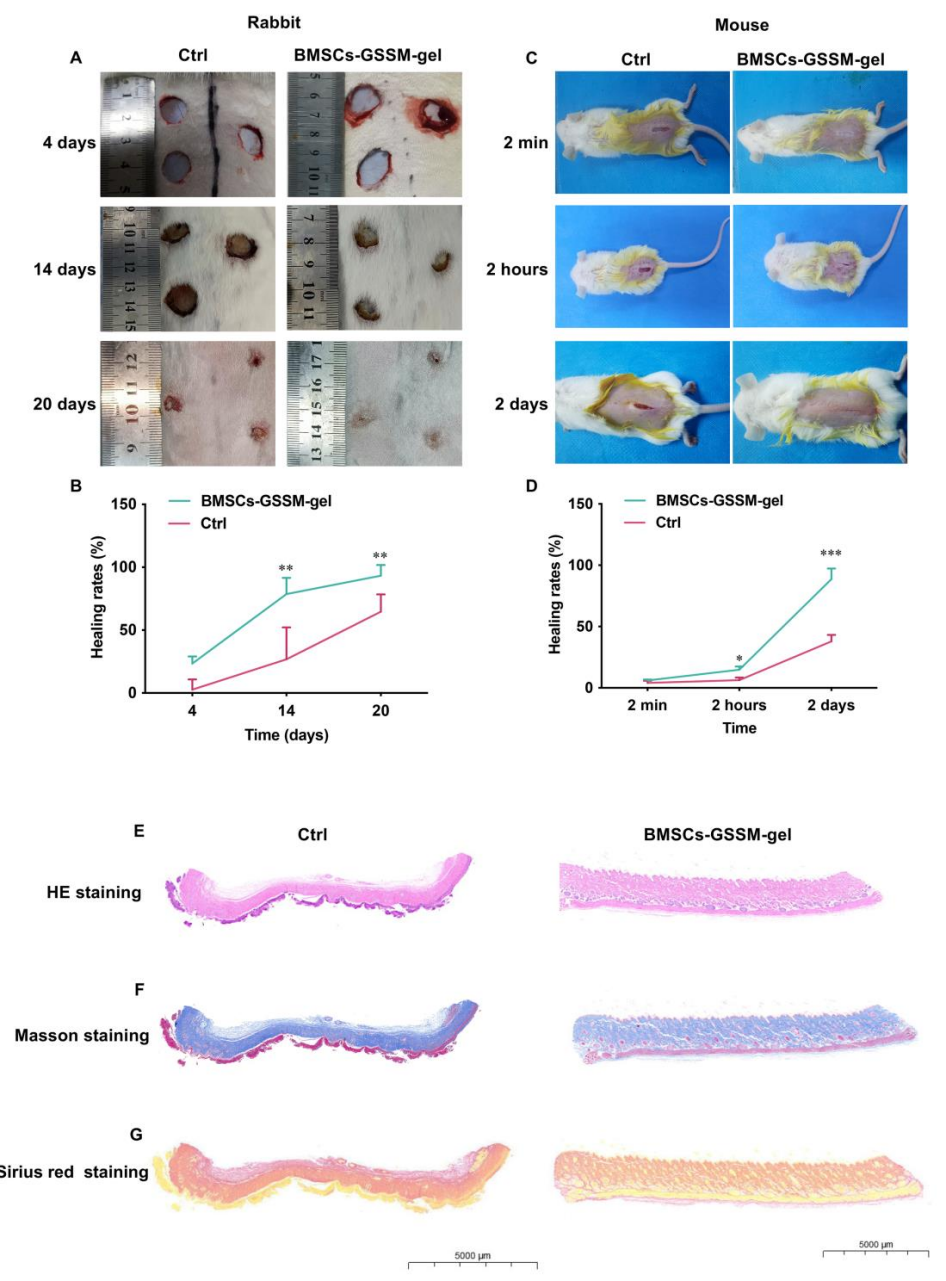
**BMSCs-GSSM-gel accelerated wound healing.** (**A**, **B**) BMSCs-GSSM-gel greatly improved skin wound healing in rabbit model (6 animals in each group). (**C**, **D**) BMSCs-GSSM-gel greatly improved skin wound healing in mouse model (9 animals in each group). (**E**) HE staining was performed to investigate skin histological changes. (**F**, **G**) Collagen deposition was evaluated with Masson’s trichrome and Sirius red staining. ^*^p < 0.05 compared with control group, ^**^p < 0.01 compared with control group, ^***^p < 0.001 compared with control group.

Two different animal models including rabbit and mice were applied to study the potential effects of BMSCs-GSSM-gel on skin wound healing. We found that wound bleeding, redness, swelling, oozing, itching, scabbing and descaling status, surface flatness and skin elasticity of the wounds were alleviated in the group treated with BMSCs-GSSM-gel on the 14th and 20th days after operation (Figure 3A, 3B). Significant wound healing rate was observed after BMSCs-GSSM-gel treatment in the mouse skin injury model (Figure 3C, 3D). The skin tissue structure was improved in the group treated with BMSCs-GSSM-gel through HE staining (Figure 3E). Meanwhile, less collagen deposition and thinner, orderly-arranged collagen structure were observed in BMSCs-GSSM-gel-treated group (Figure 3F, 3G), suggesting that BMSCs-GSSM-gel might inhibit fibrosis of skin tissue.

### The hemostatic effect of BMSCs-GSSM-gel was evaluated

**Figure 4:**
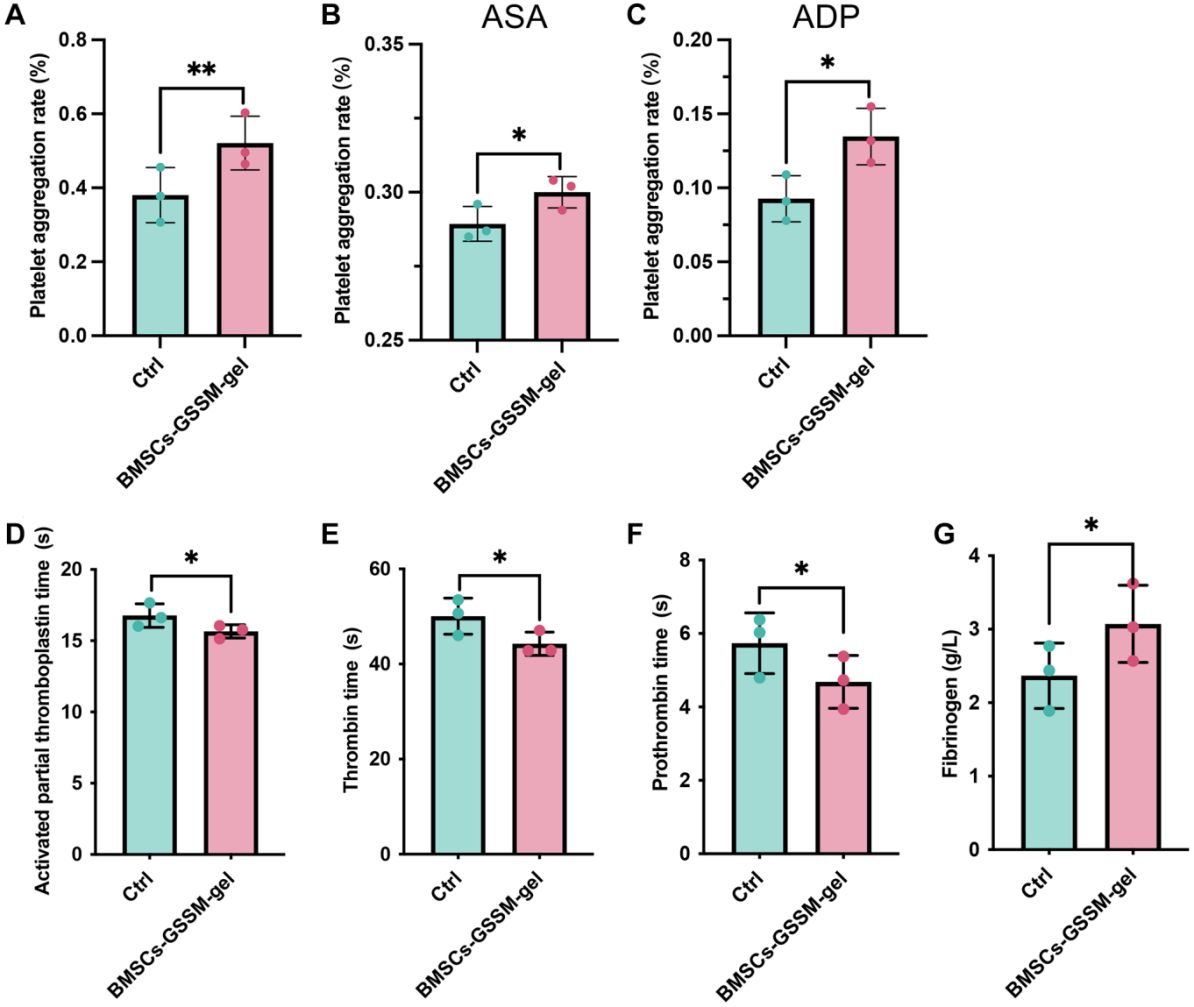
**The hemostatic effect of BMSCs-GSSM-gel was evaluated.** (**A**–**C**) The influence of BMSCs-GSSM-gel on platelet aggregation rate was investigated on the condition of ASA, ADP or not. (**D**–**G**) The levels of activated partial thromboplastin time, thrombin time, prothrombin time, and fibrinogen were evaluated. ^*^p < 0.05 compared with control group, ^**^p < 0.01 compared with control group.

The influence of BMSCs-GSSM-gel on the hemostatic effect of rabbit arterial blood was investigated. The blood samples were treated with acetylsalicylic acid (ASA), adenosine diphosphate (ADP) or not, and platelet aggregation rate was analyzed. The platelet aggregation rate was greatly increased in the BMSCs-GSSM-gel treated rabbits (Figure 4A–4C). In addition, the levels of activated partial thromboplastin time, thrombin time, and prothrombin time were significantly suppressed in the blood of BMSCs-GSSM-gel treated rabbits (Figure 4D–4F) compared with control group. However, BMSCs-GSSM-gel administration markedly elevated the level of fibrinogen compared with control group (Figure 4G).

### Target molecules were validated with transcriptome sequencing and western blotting

**Figure 5:**
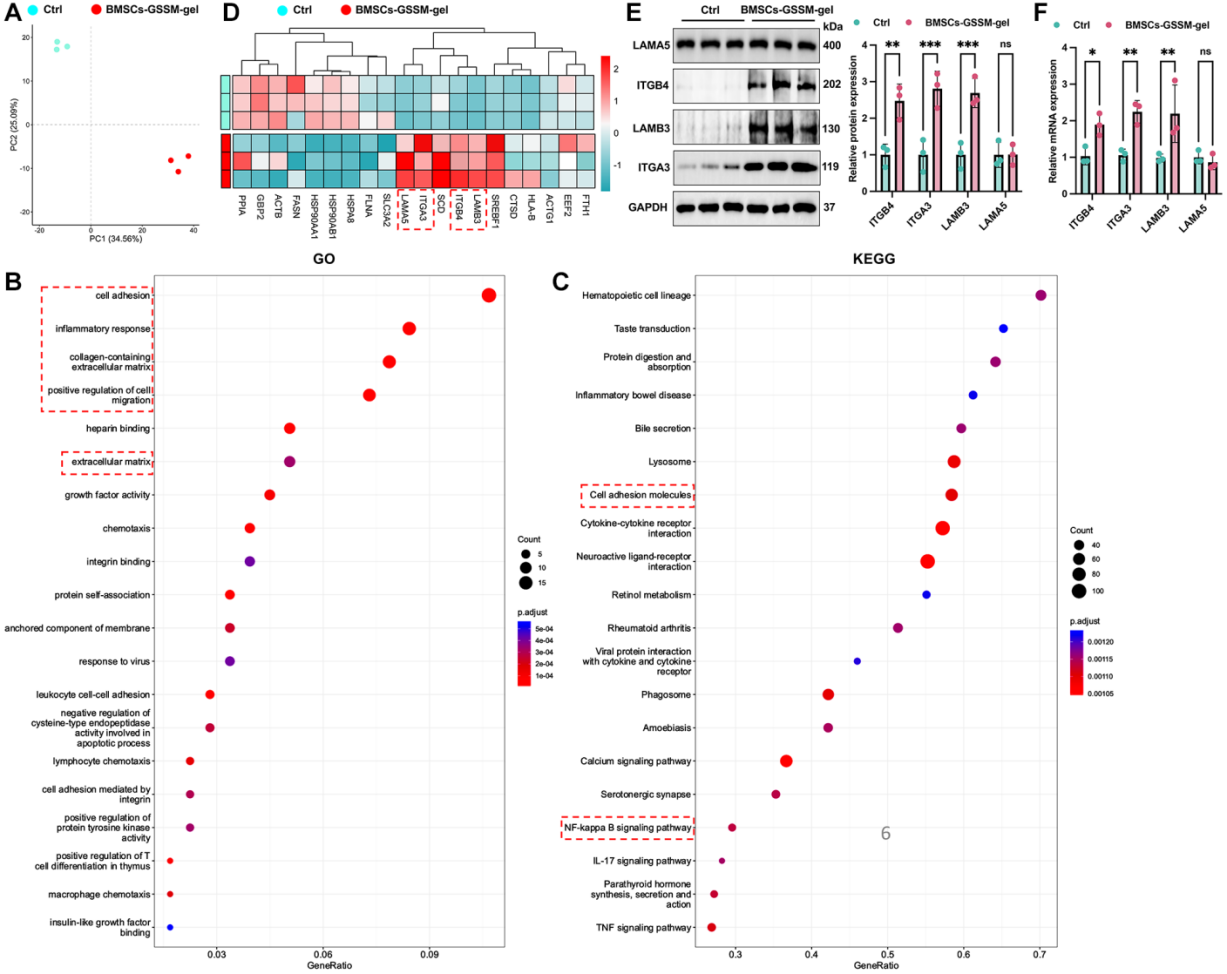
**Target molecules were validated with transcriptome sequencing and western blotting.** (**A**) PCA analysis revealed that samples within the same group clustered together. (**B**, **C**) GO and KEGG analysis was performed to find potential targeting genes and pathways. (**D**) Top 20 differential genes between the two groups were presented. (**E**, **F**) The expression of ITGB4, ITGA3, LAMB3, and LAMA5 was detected with western blotting and RT-PCR. ^**^p < 0.01 compared with control group, ^***^p < 0.001 compared with control group.

Transcriptome analysis was conducted to explore the underlying mechanisms of function. Post quality control and data processing, PCA analysis revealed that samples within the same group clustered together, distinctly separating the two groups (Figure 5A). The GO analysis indicated that the BMSCs-GSSM-gel treatment significantly impacted several genes associated with cell adhesion, inflammatory response, collagen-containing extracellular matrix, and the positive regulation of cell migration (Figure 5B). Furthermore, KEGG pathway analysis identified the top twenty pathways for affected genes, notably the cell adhesion molecules and the NF-kappa B signaling pathway, both critical in skin wound healing (Figure 5C).

Next, we focused on the differential gene expression in the skin healing model after treatment with BMSCs-GSSM-gel or not. Top 20 differential genes between the two groups were presented in Figure 5D. Furthermore, we validated the influence of BMSCs-GSSM-gel on the expression of ITGB4 [16], ITGA3 [17, 18], LAMB3 [19], and LAMA5 [20, 21], pivotal in skin wound healing. The treatment significantly upregulated the protein expression of ITGB4, ITGA3, and LAMB3 compared to the control group (Figure 5E, 5F), while LAMA5 showed no notable change.

### BMSCs-GSSM-gel promoted cell proliferation and migration of Hacat cells and fibroblasts

**Figure 6:**
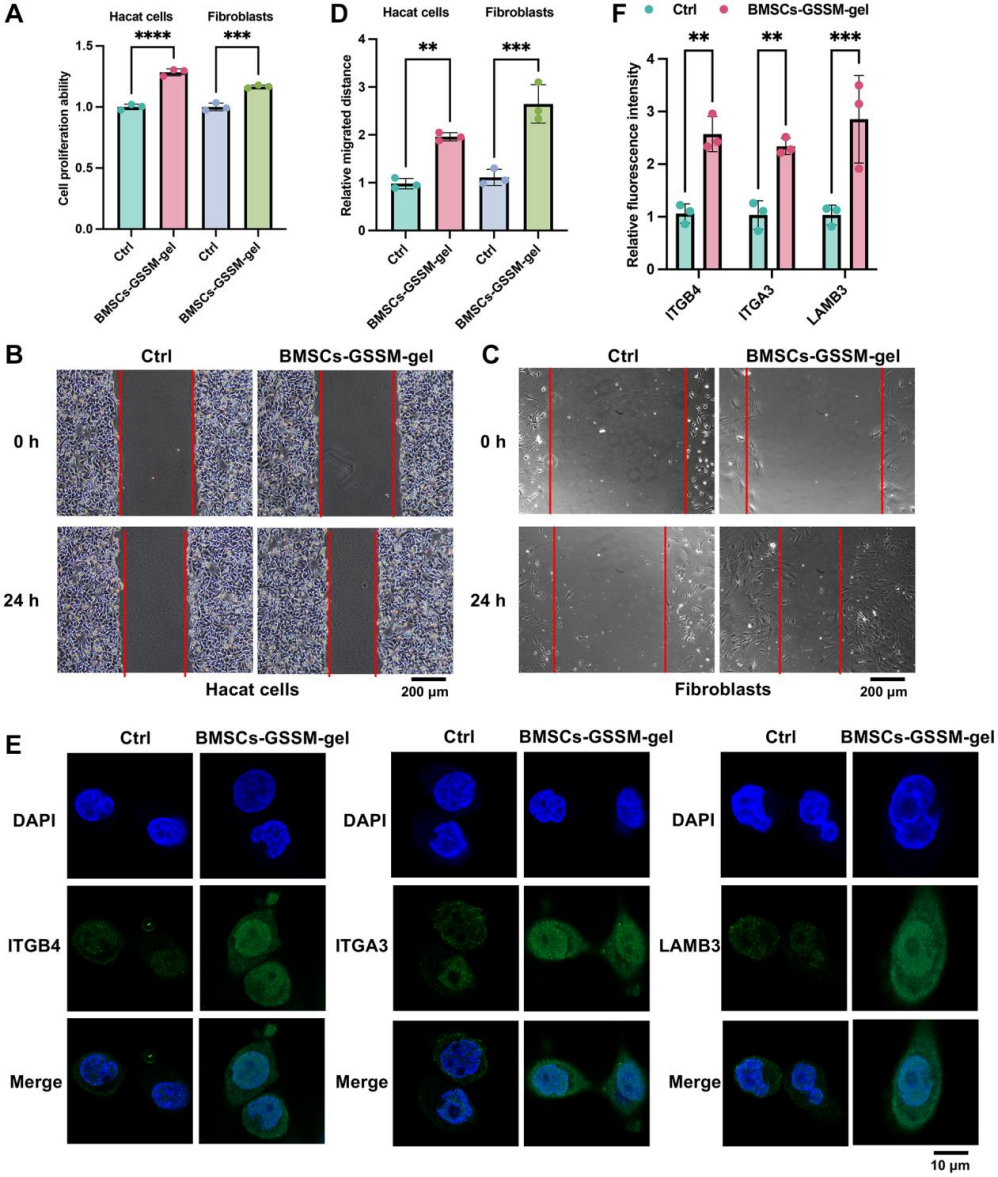
**BMSCs-GSSM-gel promoted cell proliferation and migration of Hacat cells and fibroblasts.** (**A**–**D**) The influence of BMSCs-GSSM-gel on cell proliferation and migration of Hacat cells and fibroblasts was investigated. (**E**, **F**) The expression of ITGB4, ITGA3, and LAMB3 in Hacat cells was measured with immunofluorescence cell staining. ^**^p < 0.01 compared with control group, ^***^p < 0.001 compared with control group.

To further assess the impact of BMSCs-GSSM-gel on skin wound healing, *in vitro* studies were conducted with Hacat cells and fibroblasts, integral to this process. We observed that BMSCs-GSSM-gel notably enhanced the proliferation and migration of both Hacat cells and fibroblasts (Figure 6A–6D). Moreover, the treatment significantly increased the expression of ITGB4, ITGA3, and LAMB3 in Hacat cells (Figure 6E, 6F), corroborating the *in vitro* findings.

## DISCUSSION

As an important material for the treatment of war wounds, wound dressings play a very important role in protecting wounds, rapidly stopping bleeding and saving the lives of the wounded [22]. For war wounds, the ideal wound dressing needs to have the following characteristics: can play a rapid hemostasis, to avoid or control wound infection, to provide a suitable healing environment for the wounded surface, to promote rapid healing without scarring, to improve the long-term prognosis, and to reduce the rate of secondary disability [23]. This biological property of salamander mucus makes it an excellent tissue adhesive with excellent medical properties. In subsequent *in vivo* tests, technicians found that the solid powder of the mucus has no side effects such as toxicity, making it the most promising natural medical biomaterial [24].

Inflammatory responses and oxidative stress generated during wound healing not only locally recruit BMSCs, but also promote wound healing by promoting cell differentiation and angiogenesis. It has been shown that MSCs promote wound healing by promoting angiogenesis, re-epithelialisation and granulation tissue formation [25]. In this research, the BMSCs were wrapped into GSSM-gel, and effective wound healing function was observed.

ADP is a very important platelet-activating substance in the body and plays an important role in platelet function. After being released by platelets, ADP can participate in inducing platelet aggregation, thereby promoting hemostasis. ASA is commonly used in clinical practice. ASA is an oral medication used to inhibit platelet aggregation and is clinically used to prevent or treat the formation of blood clots [26]. In this research, we demonstrated that BMSCs-GSSM-gel could greatly promote the platelet aggregation rate, whether or not ADP or ASA was added (Figure 4A–4C).

Through transcriptome sequencing analysis, ITGB4, ITGA3, and LAMB3 were found to be highly expressed in the BMSCs-GSSM-gel-treated animals (Figure 5D). Previous studies have reported that ITGA3 inhibits directional migration and wound re-epithelialization in the skin [17]. In contrast, other research suggests that ITGA3 knockout impairs wound angiogenesis *in vivo* by altering the expression of pro-angiogenic factors in keratinocytes [27]. Our results suggest that ITGA3 may play a promotive role in skin wound healing (Figure 5E). ITGB4, a structural adhesion molecule, is crucial in maintaining the integrity of airway epithelial cells, and its upregulation enhances wound repair and antioxidative capabilities [16]. Additionally, gentamicin-induced upregulation of LAMB3 has shown improved healing in junctional epidermolysis bullosa patients with nonsense mutations [19]. Our findings align with previous research, indicating that both ITGB4 and LAMB3 may accelerate wound healing.

In this research, salamander skin mucus was used as gel matrix, and BMSCs were loaded into gel matrix, which is the first giant salamander-related gel product. We believe that BMSCs-GSSM-gel may have good application prospects in the field of skin injury repair, including skin hemostasis, radiation-induced skin injury, and infectious trauma. However, this study still has the following limitations. (1) The specific functional mechanism of BMSCs-GSSM-gel in promoting skin wound healing and the signaling pathways it regulates are still unclear. (2) The application of BMSCs-GSSM-gel needs to be further validated in the clinic.

## CONCLUSIONS

BMSCs-GSSM-gel was prepared using secretions from giant salamander skin and BMSCs. The study effectively demonstrates the multifaceted therapeutic potential of BMSCs-GSSM-gel in skin wound healing in rabbit and mouse models. BMSCs-GSSM-gel also demonstrated a strong hemostatic effect, increasing platelet aggregation and regulating coagulation time markers. These findings suggest its potential as a valuable tool in advanced wound care, particularly in the context of traumatic or complex wounds.

## References

[1] He Y, Yang W, Zhang C, Yang M, Yu Y, Zhao H, Guan F, Yao M. ROS/pH dual responsive PRP-loaded multifunctional chitosan hydrogels with controlled release of growth factors for skin wound healing. Int J Biol Macromol. 2024; 258:128962. 10.1016/j.ijbiomac.2023.128962 38145691

[2] Polaka S, Pawar B, Vasdev N, Tekade RK. Development and biological evaluation of smart powder bandage for wound healing and dressing applications. Int J Biol Macromol. 2024; 258:129044. 10.1016/j.ijbiomac.2023.129044 38154708

[3] Sun J, Geng X, Guo J, Zang X, Li P, Li D, Xu C. Proteomic analysis of the skin from Chinese fire-bellied newt and comparison to Chinese giant salamander. Comp Biochem Physiol Part D Genomics Proteomics. 2016; 19:71–7. 10.1016/j.cbd.2016.06.004 27343457

[4] Geng X, Wei H, Shang H, Zhou M, Chen B, Zhang F, Zang X, Li P, Sun J, Che J, Zhang Y, Xu C. Proteomic analysis of the skin of Chinese giant salamander (Andrias davidianus). J Proteomics. 2015; 119:196–208. 10.1016/j.jprot.2015.02.008 25725404

[5] Li W, Wang M, Wang S, Wang X, Avila A, Kuang X, Mu X, Garciamendez CE, Jiang Z, Manríquez J, Tang G, Guo J, Mille LS, et al. An Adhesive Bioink toward Biofabrication under Wet Conditions. Small. 2023; 19:e2205078. 10.1002/smll.202205078 36587991 PMC10960222

[6] Li X, Li YC, Chen M, Shi Q, Sun R, Wang X. Chitosan/rectorite nanocomposite with injectable functionality for skin hemostasis. J Mater Chem B. 2018; 6:6544–9. 10.1039/c8tb01085d 32254862

[7] Liu Y, Li Y, Shang H, Zhong W, Wang Q, Mequanint K, Zhu C, Xing M, Wei H. Underwater instant adhesion mechanism of self-assembled amphiphilic hemostatic granular hydrogel from *Andrias davidianus* skin secretion. iScience. 2022; 25:105106. 10.1016/j.isci.2022.105106 36185384 PMC9519738

[8] Mu L, Tang J, Liu H, Shen C, Rong M, Zhang Z, Lai R. A potential wound-healing-promoting peptide from salamander skin. FASEB J. 2014; 28:3919–29. 10.1096/fj.13-248476 24868009 PMC5395725

[9] Dama G, Du J, Zhu X, Liu Y, Lin J. Bone marrow-derived mesenchymal stem cells: A promising therapeutic option for the treatment of diabetic foot ulcers. Diabetes Res Clin Pract. 2023; 195:110201. 10.1016/j.diabres.2022.110201 36493913

[10] Zheng L, Gong H, Zhang J, Guo L, Zhai Z, Xia S, Hu Z, Chang J, Jiang Y, Huang X, Ge J, Zhang B, Yan M. Strategies to improve the therapeutic efficacy of mesenchymal stem cell-derived extracellular vesicle (MSC-EV): a promising cell-free therapy for liver disease. Front Bioeng Biotechnol. 2023; 11:1322514. 10.3389/fbioe.2023.1322514 38155924 PMC10753838

[11] Xu JH, Xu SQ, Ding SL, Yang H, Huang X, Shi HF. Bone marrow mesenchymal stem cells alleviate the formation of pathological scars in rats. Regen Ther. 2022; 20:86–94. 10.1016/j.reth.2022.03.004 35509267 PMC9048073

[12] Wu D, Chang X, Tian J, Kang L, Wu Y, Liu J, Wu X, Huang Y, Gao B, Wang H, Qiu G, Wu Z. Bone mesenchymal stem cells stimulation by magnetic nanoparticles and a static magnetic field: release of exosomal miR-1260a improves osteogenesis and angiogenesis. J Nanobiotechnology. 2021; 19:209. 10.1186/s12951-021-00958-6 34256779 PMC8278669

[13] Dash BC, Korutla L, Vallabhajosyula P, Hsia HC. Unlocking the Potential of Induced Pluripotent Stem Cells for Wound Healing: The Next Frontier of Regenerative Medicine. Adv Wound Care (New Rochelle). 2022; 11:622–38. 10.1089/wound.2021.0049 34155919

[14] Edwards SD, Hou S, Brown JM, Boudreau RD, Lee Y, Kim YJ, Jeong KJ. Fast-Curing Injectable Microporous Hydrogel for *In Situ* Cell Encapsulation. ACS Appl Bio Mater. 2022; 5:2786–94. 10.1021/acsabm.2c00214 35576622 PMC9290187

[15] Gopalakrishnan Usha P, Jalajakumari S, Sheela UB, Mohan D, Berry C, Tripathi A, Thankappan Nair ST. Engineering cartilage graft using mesenchymal stem cell laden polyacrylamide-galactoxyloglucan hydrogel for transplantation. J Biomater Appl. 2021; 36:541–51. 10.1177/08853282211019521 34018854

[16] Liu C, Liu HJ, Xiang Y, Tan YR, Zhu XL, Qin XQ. Wound repair and anti-oxidative capacity is regulated by ITGB4 in airway epithelial cells. Mol Cell Biochem. 2010; 341:259–69. 10.1007/s11010-010-0457-y 20364299

[17] Margadant C, Raymond K, Kreft M, Sachs N, Janssen H, Sonnenberg A. Integrin alpha3beta1 inhibits directional migration and wound re-epithelialization in the skin. J Cell Sci. 2009; 122:278–88. 10.1242/jcs.029108 19118220

[18] Reynolds LE, Conti FJ, Silva R, Robinson SD, Iyer V, Rudling R, Cross B, Nye E, Hart IR, Dipersio CM, Hodivala-Dilke KM. alpha3beta1 integrin-controlled Smad7 regulates reepithelialization during wound healing in mice. J Clin Invest. 2008; 118:965–74. 10.1172/JCI33538 18246199 PMC2215730

[19] Kwong A, Cogan J, Hou Y, Antaya R, Hao M, Kim G, Lincoln V, Chen Q, Woodley DT, Chen M. Gentamicin Induces Laminin 332 and Improves Wound Healing in Junctional Epidermolysis Bullosa Patients with Nonsense Mutations. Mol Ther. 2020; 28:1327–38. 10.1016/j.ymthe.2020.03.006 32222156 PMC7210719

[20] Sampaolo S, Napolitano F, Tirozzi A, Reccia MG, Lombardi L, Farina O, Barra A, Cirillo F, Melone MAB, Gianfrancesco F, Iorio GD, Esposito T. Identification of the first dominant mutation of LAMA5 gene causing a complex multisystem syndrome due to dysfunction of the extracellular matrix. J Med Genet. 2017; 54:710–20. 10.1136/jmedgenet-2017-104555 28735299

[21] Diao B, Sun C, Yu P, Zhao Z, Yang P. LAMA5 promotes cell proliferation and migration in ovarian cancer by activating Notch signaling pathway. FASEB J. 2023; 37:e23109. 10.1096/fj.202300306R 37527216

[22] Akhtari N, Ahmadi M, Kiani Doust Vaghe Y, Asadian E, Behzad S, Vatanpour H, Ghorbani-Bidkorpeh F. Natural agents as wound-healing promoters. Inflammopharmacology. 2024; 32:101–25. 10.1007/s10787-023-01318-6 38062178

[23] Zhuo S, Liang Y, Wu Z, Zhao X, Han Y, Guo B. Supramolecular hydrogels for wound repair and hemostasis. Mater Horiz. 2024; 11:37–101. 10.1039/d3mh01403g 38018225

[24] Dang R, Chen L, Sefat F, Li X, Liu S, Yuan X, Ning X, Zhang YS, Ji P, Zhang X. A Natural Hydrogel with Prohealing Properties Enhances Tendon Regeneration. Small. 2022; 18:e2105255. 10.1002/smll.202105255 35304821

[25] Zhang Z, Li Z, Wang Y, Wang Q, Yao M, Zhao L, Shi J, Guan F, Ma S. PDGF-BB/SA/Dex injectable hydrogels accelerate BMSC-mediated functional full thickness skin wound repair by promoting angiogenesis. J Mater Chem B. 2021; 9:6176–89. 10.1039/d1tb00952d 34297017

[26] Cattaneo M. Bleeding manifestations of congenital and drug-induced defects of the platelet P2Y12 receptor for adenosine diphosphate. Thromb Haemost. 2011 (Suppl 1); 105:S67–74. 10.1160/THS10-11-0742 21479342

[27] Mitchell K, Szekeres C, Milano V, Svenson KB, Nilsen-Hamilton M, Kreidberg JA, DiPersio CM. Alpha3beta1 integrin in epidermis promotes wound angiogenesis and keratinocyte-to-endothelial-cell crosstalk through the induction of MRP3. J Cell Sci. 2009; 122:1778–87. 10.1242/jcs.040956 19435806 PMC2684832

